# Identifying Elements of Patient-Centered Care in Underserved Populations: A Qualitative Study of Patient Perspectives

**DOI:** 10.1371/journal.pone.0126708

**Published:** 2015-05-19

**Authors:** Sheela Raja, Memoona Hasnain, Tracy Vadakumchery, Judy Hamad, Raveena Shah, Michelle Hoersch

**Affiliations:** 1 Department of Pediatric Dentistry, University of Illinois at Chicago, Chicago, IL, United States of America; 2 Department of Family Medicine, University of Illinois at Chicago, Chicago, IL, United States of America; 3 University of Illinois at Chicago, Chicago, IL, United States of America; 4 Office on Women’s Health, Region V, U.S. Department of Health and Human Services, Chicago, IL, United States of America; University of California San Diego, UNITED STATES

## Abstract

Patient-centered care is an important goal in the delivery of healthcare. However, many patients do not engage in preventive medical care. In this pilot study, we conducted twenty in depth, semi-structured qualitative interviews at the University of Illinois at Chicago Health Sciences campus in a four month time frame. Many patients were underserved and underinsured, and we wanted to understand their experiences in the healthcare system. Using content analysis, several themes emerged from the interview data. Participants discussed the need for empathy and rapport with their providers. They identified provider behaviors that fostered a positive clinical relationship, including step-by step explanations of procedures, attention to body language and clinic atmosphere, and appropriate time management. Participants identified cost as the most common barrier to engaging in preventive care and discussed children and social support as motivating factors. A long-term relationship with a provider was an important motivator for preventive care, suggesting that the therapeutic alliance was essential to many patients. Conversely, many participants discussed a sense of dehumanization in the healthcare system, reporting that their life circumstances were overlooked, or that they were judged based on insurance status or ethnicity. We discuss implications for provider training and healthcare delivery, including the importance of patient-centered medical homes.

## Introduction

Patient-centered care has become an important goal in healthcare delivery. The Institute of Medicine defines patient-centered care as “providing care that is respectful of and responsive to individual patient preferences, needs, and values, and ensuring that patient values guide all clinical decisions.”[1] The Kalamazoo Consensus Statement emphasizes that a strong alliance between the patient and provider is essential in all phases of treatment. For this relationship to be successful, providers must understand both the patient’s subjective experience of his or her illness and the patient’s own health-related values and goals. The provider should also involve the patient in collaborative decision making, and not simply provide directive instruction.[2]- A systemic review of communication studies that involved neutral observers of healthcare encounters (using either direct observation or video/audio tape analysis) found the following providers behaviors were associated with increased patient satisfaction and, in some cases, greater adherence to treatment recommendations: empathy, courtesy, friendliness, reassurance, support, encouragement of patient questions, providing explanations, and giving positive reinforcement.[3] Overall, patient-centered care has been associated with higher levels of patient satisfaction, improved health outcomes and lower healthcare costs, the three key elements of the “Triple Aim” for optimal healthcare services.[4]

### Theoretical/conceptual framework

Some literature suggests that the concept of cultural competence is also a component of patient-centered care. Cultural competence requires providers to appreciate and respect the patient’s individual viewpoint, and encourages an awareness of health disparities and discrimination.[4] Cultural understanding requires professionals to look at their biases, challenge their assumptions, know people beyond labels, and develop a far greater capacity for compassion and respect. A lack of cultural understanding may negatively impact healthcare visits. For example, there is evidence that when the provider and the patient are of different ethnic backgrounds (race-discordant), providers spend less time with patients; additionally, providers may use fewer patient-centered strategies when talking to ethnic minority patients.[4] Awareness of a particular culture can provide a useful “short cut” to understanding the general values, beliefs, and behaviors of an individual, but there is a danger that it can stereotype that individual. The result is that individual needs are not identified and met. When exploring aspects of patient-centered care, it is important for providers to consider how underserved and underinsured/uninsured populations may experience medical visits. These patients may have more difficulty accessing healthcare,[5] and those with access to care may not be engaged in preventive health services.[6]

In terms of preventive care, the literature suggests that patients’ motivations are often complex. Psychological theories of health behavior change emphasize that patients must: 1) believe that a particular behavior is important (e.g., obtaining a mammogram, quitting smoking), 2) feel they have the skills to attempt behavior change, and 3) have social and environmental support to change the behavior.[7] However, there are widespread disparities in preventive care utilization.[8] For example, minority populations are less likely to obtain mammograms, Papanicalou tests, influenza vaccines, cardiovascular procedures, and prenatal care.[9] Lower socio-economic status is also associated with lower levels of prenatal care, mammography, and colorectal cancer screening.[10] Finally, intermittent health insurance coverage is also associated with lower use of preventive services over a period of years (even when participants regain their insurance).[11] Factors such as cost, access to care, beliefs about the importance of preventive care, and environmental factors all play a role in these disparities.[9]

An important aspect of both patient-centered care and cultural competence is understanding how patients themselves, in their own words, experience medical treatment. Qualitative techniques are particularly useful in healthcare communication research because they can explore relationships between attitudes, behaviors, and experiences.[12] Qualitative research can also help broaden theory and generate new ideas. This pilot project involved in-depth interviews focused on understanding how patients at an urban, University-affiliated medical center experienced medical care. We were particularly interested in what factors facilitated engagement or disengagement in the healthcare system, and if our patient population placed a high value on the principles of patient-centered care. We also wanted to understand patients’ motivation for engaging in preventive care.

## Methods

### Qualitative interviews

Twenty participants completed semi-structured, qualitative interviews. All interviews took place in a private office with the principal investigator (PI), a behavioral scientist with training in health psychology. Interviews took approximately 45 minutes to one hour to complete. All participants received a $20 grocery store gift card to compensate them for their time.

Open-ended questions focused on where participants obtained their healthcare and if they visited a physician regularly. Participants were asked to reflect on their most positive or negative healthcare visits. The interviewer also probed for details about what—aside from the actual medical diagnosis—made specific encounters positive or negative. Participants were asked to think about ways in which healthcare services in general could better meet the needs of patients. Participants were not limited to reflecting on their experiences at the University health clinics. Many participants discussed treatment experiences in various settings and area clinics. Participants were also asked what motivated them to engage in preventive medical care. If participants discussed barriers to care, they were asked to reflect on how various demographic issues (e.g., ethnicity, insurance status) may have impacted specific healthcare interactions. Although the interviewer asked broad questions about these topic areas, the interview was largely participant-driven and interviewees decided how much time to focus on each topic.

This project was approved by the University of Illinois at Chicago Institutional Review Board (IRB). Participants signed an informed consent form advising them that: they could stop the interview at any time, they could refuse to answer any question without penalty, and their participation did not influence their ability to receive healthcare at the University clinics. Interviews were completely confidential. Participants agreed to have their interviews audiotaped for later transcription. Transcriptions did not include any identifying information; references to proper names or names of specific healthcare providers were omitted during transcription. All audio recordings were destroyed after transcription.

### Study setting

The University of [deleted to ensure blind review] health sciences campus is located on the near west side of [deleted to ensure blind review]. The campus clinics and hospital serve patients who are largely uninsured or underinsured. Sixty-five percent of the patient population is female and 35% are male. Many patients are underserved ethnic minorities, with a patient population that is approximately 10% Hispanic and 34% African American. The dental clinics, also located on the health sciences campus, serve a patient population that is approximately 25% Hispanic and 25% African American.

In general, all patients require a referral from a family physician in order to see a specialist. The copayment amount is based on insurance status, and can range from approximately $3.90 for Medicaid and to $40–60 for other managed care plans. The average copayment is approximately $20, and there are no ceilings for copays. In general, patients seek care from their primary care physician first because specialty care often involves additional copayments and waiting times. The majority of patients seeking dental care at the University clinics are eligible for Medicaid benefits or pay out of pocket for dental procedures that are not covered.

### Study participants

Participants were recruited using two methods: 1) As part of a larger survey-based study on experiences in the healthcare system, patients in the waiting room at the University of [deleted to ensure blind review] dental and family medicine outpatient clinics were given an optional locator form to fill out if they wanted to be contacted about future in-depth interviews. Patients who agreed to follow up interviews were contacted by phone to schedule an in-depth, one-on-one interview. 2) We also posted IRB-approved fliers around the health science campus to invite patients to discuss their experiences in the healthcare system. Patients who were interested in being interviewed contacted our study staff. Based on the established guidelines for conducting qualitative interview studies, our target sample size was twenty patients to achieve data saturation.[13]

Participants ranged in age, ethnicity, and in where they received medical care. Because of our recruitment approach (recruiting at University outpatient clinics as well as posting fliers), participants received healthcare services at University clinics as well as other local clinics and hospitals. Eighteen of our participants were female (six Hispanic, five African American, and seven White participants). Two of our participants were White males. Participants ranged in age from 21 to 74 years old.

### Analysis

We analyzed the interviews using content analysis, as outlined in Hancock.[14] The research team consisted of the principal investigator and four research assistants. The team was ethnically and socioeconomically diverse. Some of the research assistants intended to pursue careers in the health sciences and others were focused public health and social work. Part of each team meeting was focused on processing our own biases and reactions to the interview materials. All the research assistants were trained in research ethics and the importance of self-reflection and care when engaging in qualitative research.

The coding scheme was created using an iterative process. The semi-structured interview questions were based on a prior knowledge of patient-provider behavior and we expected that patients would discuss issues such as empathy, rapport, step-by step explanations, and time management when discussing healthcare experiences. However, the specific categories and larger phenomena that emerged from these interviews followed from an inductive process, meaning the research team read the interviews without any preset coding scheme.[15] Initially, the PI and research assistants read through five transcripts and made notes about general themes that emerged in these interviews. The team then met to create an initial coding scheme. The themes of empathy and rapport emerged quickly because they were anticipated.

Each team member individually read through another five transcripts to add to and refine the coding scheme. We met again as a team to create a set of categories for our coding scheme. A strong theme of dehumanization emerged after further team discussion and in-depth examination of the transcripts. Similarly, the importance of a long-term relationship with a provider emerged when examining the theme of preventive care. Based on group discussion, and eventually group consensus, the coding scheme was divided into major and minor categories, and eventually, larger themes. As we created and revised our coding scheme, we did not feel that the common emerging themes differed between men and women. As such, we retained all 20 interviews for analysis.

Each transcript was analyzed by two team members. Discrepancies that arose in coding were discussed in team meetings and resolved by group discussion. The final coding of each interview was entered into Atlas.ti. Finally, we examined the final categories for larger phenomena and the team reached consensus on how phenomena fit into the larger framework of patient-centered care.[16]

## Results

**Fig 1:**
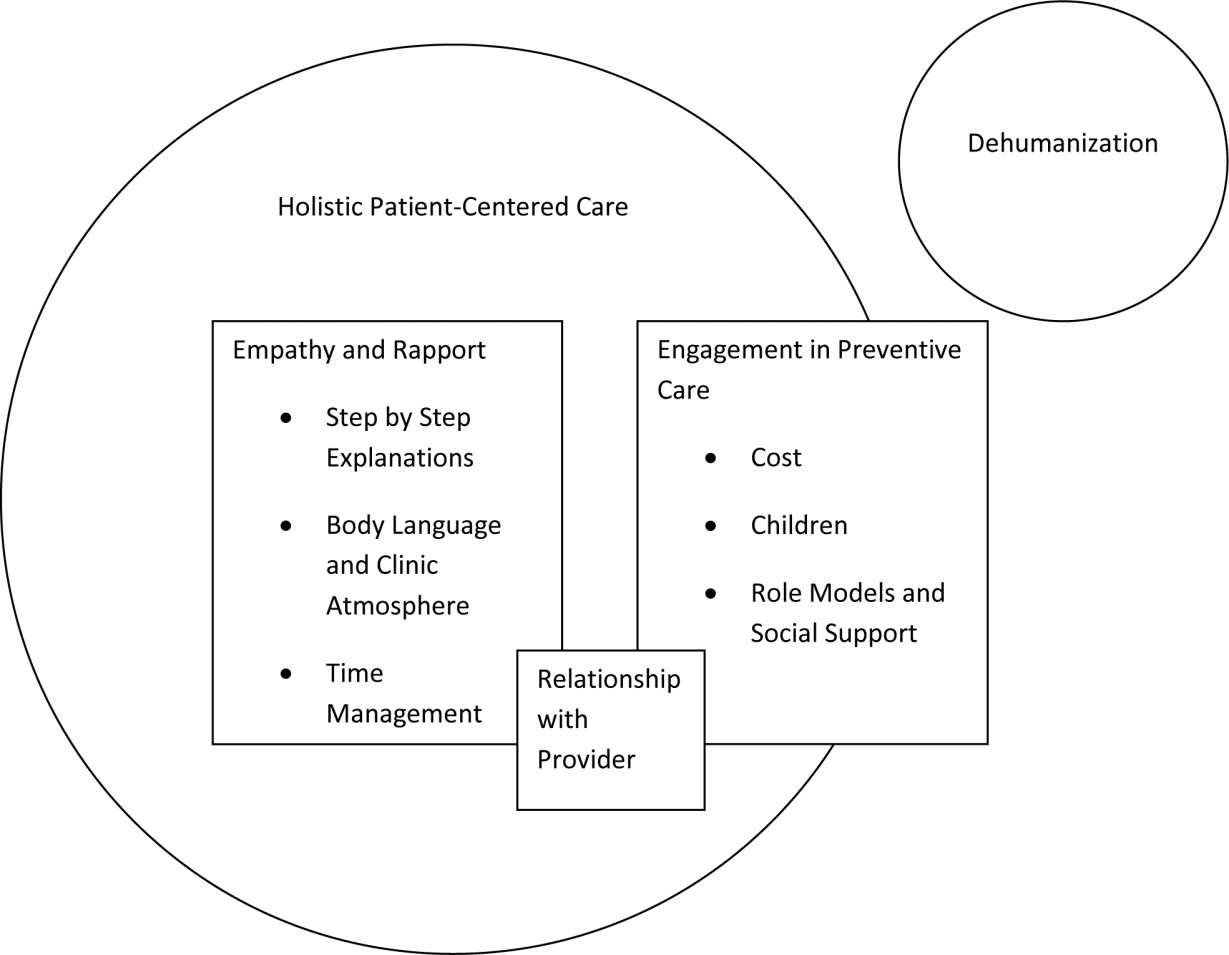
Relationship between dehumanization and the elements of holistic patient care based on content analysis of interview themes.

Participants reported that compassion, empathy, and rapport were the cornerstones of a positive clinical experience. They went on to discuss specific behavioral strategies by which doctors could create positive relationships with their patients. These included providing step-by- step explanations and frequent opportunities for patient questions, the importance of paying attention to body language and creating a positive clinic atmosphere, avoiding clinical jargon, and managing time appropriately. In terms of motivation for preventive care, patients frequently discussed cost as a major barrier. Participants reported that children, role models and social support, disease prevention, and a strong relationship with a steady provider were motivators for preventive care. An overarching phenomenon that participants described was the desire for holistic or patient-centered healthcare, an approach that focuses on the entire individual, and not just the immediate symptoms. In contrast to holistic care, many participants discussed a feeling of dehumanization that sometimes occurs in the healthcare system. Fig 1 provides an overview of the relationship between themes that emerged from our analysis.

### Compassion, empathy, and rapport

Many participants had general advice on how providers could create strong relationships with patients.

Just probably soothing voice, chatting with them…Kind of, you know, getting to know a relationship, and if they have issues. A lot of people going to doctors do have mental issues (62 year old, White female)

Talk to them, find out what is going on. If they are having problems with pain, it may not be all the pain, it may be something else or maybe a hidden symptom with that pain. But if you don’t find out what that symptom is you are not going to cure the pain. (46 year old, White male)

Some participants mentioned that they feel the connection with their providers is not one-sided and that part of connection involves knowing their providers as people. This helped participants feel more like equals in their interactions.

I know that he’s got four children. I know that his wife has rheumatoid arthritis. He knows a lot about my life…When he comes in, he’ll say, “Hey, are you still working with that family that we talked about last time?” Just little things. Little vignettes. But that really helps bridge the gap a little bit. (74 year old, White male)

Some participants talked about positive experiences in large, teaching hospitals.

She's a rheumatologist…I feel no judgment; I feel she treats me with care and compassion. …I feel I can ask her anything and I can say anything to her and she'll give me a professional response. She'll be very compassionate and caring; she's being a good listener. I think those are some of the primary attributes she possesses and same here with the doctors I've encountered here at the clinic. They seem to ask questions that they really want to know the answer to. (52 year old, White female)

However, not all patients agreed. Some feared that it would be difficult to establish rapport at large teaching clinics.

In the past if I took them [my kids] to the [community] provider that we’ve been seeing for ten years, once I had one positive diagnosis, I could just call up and say, “I’ve got another [child sick with strep],” and they’d phone in a prescription for me because we’ve had this rapport…Whereas [at my university-based teaching clinic], I had to bring my children down here…My fear is I don’t know how I’m going to gain that rapport with a physician here because it’s such a large practice (39 year old, White female)

Several participants also discussed situations where they felt they did not experience a sense of rapport with their providers.

I always felt like they didn’t ask enough questions and they didn’t tend to your needs. To me, it felt like a routine, robotic-type thing. It was like, “Okay. What are you here for? This is what you need.” (43 year old, African American female)

Like [a large area hospital] for a while had a big thing, “Patients First”. They laid it on so thick that it was actually a turn off…It’s almost like theater, bad theater, like you really give a shit about me because you don’t…I wish the first time he met me he wouldn’t have just gone through like the rudimentaries, like family history of diabetes, heart disease, cancer…The first time I met you [ask], “What made you come to me? Who was taking care of you before? I know that you’re here today because of this. But you’re my first-time patient. I would like to establish a relationship. (65 year old, White female)

One participant talked about how demographic differences between patients and providers may hinder forming an alliance.

Some other people, like these shelter[ed] White girls, who go to college, who don’t have the life experiences, they don’t know. How could they possibly understand? You’re going to tell me you understand what it’s like to be homeless. (41 year old, White female)

### Step-by-step explanations

Many patients felt that providers should pay more attention to providing patients with an overview of procedures and giving them clear expectations about follow-up procedures. Many participants reported this was a key factor in distinguishing between their positive and negative interactions with providers. They discussed the importance of providers giving them an overview of lab results, the appointment flow, and a realistic expectation of pain both during and after procedures.

They explain everything to you before they do anything….They tell you what they're going to do, you know it's going to hurt a little bit and stuff like this, they just tell you everything in general. And I like that. (63 year old, African American female)

I want every report of any test that I have…I can read and I can tell what’s on that report. I don’t want some doctor saying, “You know, your test was all right.” No, I want to know what my blood test was. I want to know what my HDL and my LDL and everything on that. (74 year old, White male)

Some patients reported experiences where clinic procedures were not clearly explained. This led to frustration and anxiety.

“I’ll have my referral coordinator do it.” He wouldn’t communicate…I’d be waiting. I’d call the office…I’m talking about a week, week and a half later…. He wouldn’t call back. …I’m going to get anxious if I’m not getting the answers and you’re not explaining it to me. (41 year old, White female)

I even had seen the doctor, they came in and didn’t ask me anything and gave him [my son] some medicine by mouth. And I said, “What is that?” And they said, “It’s a steroid.” And they didn’t ask me. They didn’t tell me. It wasn’t even the doctor. It was a nurse. (39 year old, White female)

Well, something really bad happened when I got out of the hospital. I was taking 275 milligrams of steroids three times a day…When I left, nobody showed me how to use insulin. …But just coming out of the hospital and bouncing off the walls from all the steroids in me, I didn’t really understand a thing that they said…and then when I got home I was really scared. (55 year old, Hispanic female)

### Paying attention to body language and clinic atmosphere

Many participants underscored the importance of non-verbal issues. This included the need for providers to pay attention to their own body language, as well as the body language of patients. Participants also paid attention to the physical details of a clinic environment. A pleasant environment made many participants feel respected and welcomed.

Sometimes you have to see how people react…Listen to patients. Listen to their cues. Watch their body language. Watch, are they leaning in? Are they sitting back? Are they guarded? Are they not looking? (41 year old, White female)

A clinic environment with amenities and distractions was also valued by participants.

When I went back, it was so long ago, when I went back there [to a teaching clinic] you had to bring your lunch and sit on a folding chair. Now they got nice a waiting room, …with [vending] machines. (68 year old, White female)

### Avoiding clinical jargon

Participants overwhelmingly discussed the importance of providers being able to express technical terms in an understandable, patient-centered manner.

A lot of the medical terms you don’t understand…I was like, “Well, what’s an ignorant murmur?” And he’s like, “Oh, it’s nothing.” He [the physician] literally kind of like, “It’s nothing for you to worry about.” And to me, a murmur is a hole in the heart. Whether it’s small or big, to me, it’s a big deal. He kind of just blew me off. (39 year old, Hispanic female)

Quit using such big words where peoples don’t understand..Talk at folks level. So they can, you know, be able to comprehend what the doctor is talking about. (50 year old, African American female)

And she’s relational. I don’t care how much she knows. If she’s going to keep it crammed up there and refer back to her studies and teach in the damn medical school, that doesn’t do anything for me. It’s like a bunch of zombies coming out of there. (65 year old, White female)

### Time management

Participants also expressed the desire to have more physician time during their appointments. Some also expressed frustration that appointments were difficult to schedule due to provider availability. Rushed appointments and difficulty reaching providers were associated with feeling devalued.

He was very much the 15 minute, I’ll call them, doctors. 15 minutes, he goes through all the paperwork, he just wants to make sure that he’s not going to be liable for anything that he, if he’s negligent. (59 year old, African American female)

When you call a new place, they’ll be like, “Well, we have an appointment a month from now…So every time you switch a doctor, you’ve got to wait. (41 year old, White female)

Participants had a clear understanding that time was a premium in medical settings. They were very appreciative of a doctor that spent extra time with them.

I called and got an appointment that day and the doctor just spent a long time with me going over my routines and asking me a lot of questions…I just wanted some medicine because I was kind of burned out at that point but it just seemed like a really good appointment. (26 year old, White female)

Many participants were sympathetic to the time pressures that healthcare professionals face.

The doctor would remind me in a nice way, not negatively, he’d say, “Now, look.” He said, “I’d love to sit here and talk to you, but we have like an eight-minute frame here…And I want to get to your medical needs.”…I think the institution needs to be a little more sensitive to that, and help these doctors a little bit because they’re under constraints. (74 year old, White male)

Participants also made a connection between multiple visits, time pressure, and financial concerns. Participants noted concerns about multiple co-pays and time off of work.

I know they are trying to get as many patients as possible in in an hour. Sometimes it is very difficult to diagnose the problem within those ten minutes …It’s not—the cookie cutter method is not—let’s try this and this and if it doesn’t work come back next week…People can’t afford to come back next week, can’t afford to get, “Oh, this medication didn’t work so let’s try this medication,” then that’s another $30. (46 year old, White male)

One participant with cancer synthesized that multiple institutional demands on her physician (at a large teaching hospital) left her feeling like she was not valued.

I called the office, talked to him. “Can’t talk to him.” “Well, I guess I’ll have to set up an appointment.” “Well, he can’t see you for another five weeks.” So I feel like this guy is not—doesn’t have the time or isn’t given the rope by whoever is the chairman there or the chief of staff.…He’s busy teaching because he’s in charge of the medical school with the students…Like it’s [scheduling appointments with patients] something he have to do, but such a waste of his time. (65 year old, White female)

### Motivation for preventive care

After discussing what went into positive and negative healthcare interactions, participants explored their motivations to engage in preventive healthcare. Although we did not directly ask patients about barriers, they spontaneously identified one major barrier—cost. Participants also discussed the children, social support, and prevention of disease as motivators for preventive care. Some participants mentioned that their relationship with their provider was major motivator to engage in preventive care.

#### Cost as a barrier

Nearly every participant mentioned the cost of healthcare.

I'm on disability…All I have is a medical card…It covers 15 minutes in going to see the doctor, a co-payment of $4.00… $4.00 is nothing, if you have $4.00 to give every time you go. But if you're a person like me that has six or seven illnesses, going to see the doctor and paying them $4.00, that adds up to $30.00, $40.00 a month,..you can't afford it. So what I've done now is I've cut down going to the doctor. I go as little as possible. (55 year old, Hispanic female)

They bother me constantly, telling me that I’m entitled to come in for all this preventative, wellness treatments. I’m not getting involved with that. I’m not going to spend a dime for this or that more than I have to…Cost is the number one factor because,…I still have to pay my deductible, and I still had to pay a co-pay for every doctor I saw…He could refer me to somebody, like an allergist there or a pulmonologist. I saw four doctors, had four visits, that’s $100 tacked onto $250, yeah, which is $350, which means I don’t think so. (65 year old, White female)

Although participants reported cost as the major barrier to care, they also noted several factors that motivated them to seek medical care.

#### Children

Several participants reported wanting to stay as healthy as possible for their children,

My kids are around, I need to be healthy for them. I need to be strong. You know, my mind needs to be clear…And if I’m not that way, who is going to help them? (31 year old, African American female)

A few participants noted that when money and time is limited, their children’s medical care takes priority.

When my son was a child, I would go more often because I wanted to make sure that I was okay as a single parent, but after he got grown, I guess I figured I can exhale. (59 year old, African American female).

#### Role models and social support

Participants also discussed the important role that friends and family play in attitudes toward preventive healthcare. Some social networks supported a healthy lifestyle and others discouraged it.

And my parents, when we were growing up, they did it [went to the doctor] on a regular basis. Basically, it is due to experience and what my parents did with us and the way they raised us….I think that’s just from the women in my family. They’re just some headstrong women. (43 year old, African American female)

I always manage to have somebody that is in some way not well, not healthy…And that absolutely does affect how I take care of myself…Smokes his cigars in the middle of the night because he kind of winds down at 4:00 am, which totally affects me. It affects how I take care of myself. (65 year old, White female)

#### Prevention of disease

Many patients discussed prevention of disease as a major motivator in seeking regular medical care.

I mean, there’s so much going on now with cancer and all that. And the faster you detect it, you know, the better chances of you overcoming, fighting that, you know, disease. (39 year old, Hispanic female)

I try to stay ahead of the game. Why have a stroke? Why reduce your quality of life if you can do it by going into the doctors and making sure you’re on top of things? …The way I’m looking at it, “Look, I have respect for myself and I want to do the best thing to keep my body and everything in shape as long as I can.” (74 year old, White male)

However, not all patients were engaged in preventive care.

Now I have high blood pressure so I have to go get that medication. And then I’m going to be on Syntheroid for the rest of my life so I do go get that…But other than that, I have to be sick, I mean, really sick to go to the doctor. (50 year old, African American female)

#### Relationship with their providers

Several participants felt that their relationship with a particular provider was an important motivator for attending regular medical care. Interestingly, this theme functioned as a link, connecting the themes of compassion, empathy, and rapport with engagement in preventive care. As such, the therapeutic alliance itself can be seen as a tool for engaging patients in preventive services. However, in large teaching clinics, these types of relationships may be rare.

Where many a doctor get in there [a large teaching hospital], they learn, and then they move or go onto their own practice or move to a different practice. And she’s been a stable doctor there…She kind of knows me medically. And I could call the office and say, “I’m D—-, I’d like to either talk to her or can she squeeze me in?” (62 year old, White female)

He was just very sensitive…I got hypertension as a result of pregnancy, and he was furious. “You need to take better care of yourself. You didn’t have hypertension before this…” And he took it personally like it was his fault almost that, and he was angry at me that I was not monitoring what I was eating…He was just was just so concerned, and he was right…He was very diligent about trying to help me overcome it. (59 year old, African American female)

### Holistic, patient-centered approach

A large, over-arching category that emerged was the importance of holistic, patient-centered care, where providers considered the totality of a patient’s physical health, their ways of coping, and their environment. Participants felt that providers must use a holistic approach to form trusting relationships and engage patients in preventive and follow-up care.

Well, he walked me through the diet. Stress…So, he did a very comprehensive job of informing me of ways to turn this around… I just would like doctors to be a lot more conscious about natural ways to help people, not just so readily to give a prescription…Half the prescriptions that I get, I don’t even fill them. (59 year old, African American female)

You’re a whole person… If you have heart trouble, it might affect your feet hurting. I don’t know if it does, but it could. I suppose if you didn’t get circulation down to them. So then you go to a foot doctor, you know, it’s just too many, I think that there was a real loss, … when they got rid of the old family doctor…Treat the whole person. **…** maybe to communicate a little more about other than the disease…Be a little more involved. (68 year old, White female)

He learns more about me than just what’s on the chart, or, you know, my cholesterol level, or, you know, whatever…I think they [doctors] have to know what’s going on in their communities…be willing to climb into that world a little bit. (74 year old, White male)

### Dehumanization

The opposite of holistic care was a feeling of dehumanization that many participants experienced in the healthcare system. Dehumanization is defined as when a person’s mind is not seen as “distinctly human.”[17] In medical settings, this occurs when the patient is seen as lacking feelings and emotions, or when they are seen as lacking agency, or the ability to make choices. ^17^ In our sample, dehumanization resulted from not feeling listened to, cared for, or seen as an entire human being in healthcare settings. In the process of dehumanization, patients may feel their needs are somehow secondary or unimportant.

For example, many participants expressed dissatisfaction that their symptoms were not being treated in the context of their specific life circumstances. One participant discussed seeking medical care after being hurt by a boyfriend.

You know, instead of recognizing, “Wow. Is everything okay? Tell me about your relationship. What is going on here?” You could tell, at least I felt that he was thinking that it was just like a girl with like too much [sexual] experience versus this could potentially be like an unhealthy situation. (27 year old, White female)

Another participant was upset that her provider assumed her illness was a result of unprotected sex.

[I had] A urinary tract infection in college. I didn't know what it was. I never had one but the doctor said something like I was having unprotected sex or something and I got really offended and then I realized that that is what normally the cause is and even though it wasn't the case with me… It's just like your case is so similar to everyone else's it can kind of feel like you're not having individualized treatment or service or whatever. (26 year old, White female)

Other participants felt their symptoms were not being taken seriously.

And I’m steady telling him something is in my throat. And to him, it’s nothing wrong with me because …there wasn’t anything wrong with my blood level. But I’m steady telling him what I feel, you know. But he don’t hear me anymore. (50 year old, African American female)

As a part of dehumanization, some participants also felt judged based on social class and ethnicity, which was tied to a lack of perceived cultural competence on the part of providers.

I think that she [the medical receptionist] automatically put me into a certain class once I said I need a letter for the utility company [saying she had a medical disability]. She went from a very friendly person to a very condemning person…She went from very nice and friendly to a bully just because she thought, “Okay, you’re in a certain class and … you’re going to give me some social proof that you’re not [of a lower class].” (59 year old, African American female)

That just because I’m here and I'm on public aid and I have a medical card, you know, you [think] "Oh, she's just on Medicaid anyway and she's just living off the system. (55 year old, Hispanic female)

She goes on to express her disappointment on the emphasis on money in the medical system.

Now everything is about money…So if you have your mind on money, there's not much attention you're giving that patient….Until you really feel like, “this is my calling, this is what I’m here for…” If you work in any kind of medical field, I mean, you're going to make money…How much money do you need? (55 year old, Hispanic female)

One participant summarized a sense of dehumanization as follows:

But I think the way that medical doctors are taught, I think they lose sight of the human a little bit. And they have more of the science in their head instead. (39 year old, White female)

## Discussion

This pilot study sought to explore patients’ perspectives regarding their experiences of clinical care in outpatient primary care settings. Although many healthcare systems rely on patient satisfaction surveys, the narratives of patients are often overlooked in healthcare administration and training. In general, our in-depth interviews supported the importance of patient-provider communication in healthcare. Participants discussed the significance of compassion, empathy, and rapport. Compassion in healthcare is generally seen as the ability to engage with patients empathically, tolerate their distress, and have a strong motivation to help. Participants discussed all of these aspects of compassion in their interviews.[18] Within this larger theme, participants discussed the importance of step-by-step explanations, body language and clinic atmosphere, and appropriate time management. Participants also discussed what motivated them to engage in preventive care. Cost was seen as a major barrier, while children, social support and role models, and disease prevention were major motivators to engage in preventive care. It is interesting that some patients spontaneously mentioned their relationship with their provider as a motivation to engage in preventive care. This underscores the importance of the patient-provider relationship. In fact, the relationship itself can be seen as a bridge. As suggested in the Kalamazoo Consensus Statement, once initial rapport is established, a quality relationship seems to be an important part of keeping patients engaged in the healthcare system.[2]

Our participants also brought up two overarching themes that we did not ask about directly in our semi-structured interview. The categories of holistic, patient-centered care, and dehumanization strongly emerged in our data analysis. The desire for providers to consider their whole lives—their medical, psychological, and environmental concerns—was extremely important to our participants. Participants also noted that a long-term relationship with a steady provider contributed to a feeling of holistic, patient-centered care. This theme complemented the finding that a strong, long-term relationship with a provider can serve a motivator to engage in preventive care. Participants also described feeling dehumanized in healthcare encounters. When patients were not treated with respect or when they felt they were judged based on socio-economic status or race, they became less likely to engage in preventive and sick care. Interestingly, patients did not generate suggestions for how providers could change their behavior to address the dehumanization that results from demographic and ethnic gaps. Perhaps this is indicative of the level of alienation that these patients were experiencing.

According to participants, providers’ use of step-by-step explanations, encouragement of patient questions, and avoidance of jargon were all ways in which the patient-provider gap could be bridged in the hopes of receiving holistic, patient-centered care. These methods to solidify the patient-provider relationship may also combat an underlying barrier to care: inadequate health literacy, especially important among underserved populations. Health literacy has been defined by the Institute of Medicine (IOM) in its 2004 report as “the degree to which individuals can obtain, process, and understand the basic health information and services they need to make appropriate health decisions.”[19] Much of the literature on health literacy has foundationally addressed patients’ roles in and the diminished patient outcomes due to low health literacy[20], yet not nearly as much attention has been paid to healthcare providers and their responsibility in mitigating this barrier to care.[21] An important aspect of effective communication between the patient and the provider is the ability for the patient to not only understand the technical language of the provider, but to also actively and critically think, inquire, and apply medical advice and protocols to his or her treatment plan. In underserved populations, patients’ limited access to health information can hinder their ability to obtain care and to understand the complexities governing the delivery of healthcare.

Underserved patients also discussed the theme of dehumanization in healthcare settings. This theme is closely related to the concept of institutionalization, which is defined as a “system of rules, beliefs, norms, and organization that can jointly generate a regularity of behavior in a social system.”[22] As medicine has become institutionalized, patient outcomes are measured in clinical and economic terms, meaning that cost-effectiveness influences treatment decisions.[23] The institutionalization of medicine has also led to lack of emphasis on outside factors that may impact health (e.g., nutrition, safety, employment, and neighborhood stress),[25] leaving marginalized people at high risk for illness and disease.[24]

From a clinical standpoint, the larger categories of holistic, patient-centered care, and dehumanization are also consistent with recent effort to establish patient-centered medical homes (PCMH) when treating underserved populations. The PCMH can help integrate patient care, foster patient-provider communication, and create linkages with other services in the community.[26] Although providers in the PCMH model report challenges such as limited time with patients, a lack of easy follow-up methods across services, and a lack of financial incentives for preventive services, this may be a promising model to begin to address the issues of underserved populations.[27]

In large, urban teaching hospitals, our findings suggest that trainings of residents and ongoing clinical education should focus on how dehumanization may hinder holistic, patient-centered care. Haque & Waytz[17] outline several factors that encourage dehumanization in medical settings. For example, medical practices tend to de-individuate patients (e.g., all patients wear standard gowns), patients have less autonomy because they are impaired (particularly during sick visits), and patients and providers are dissimilar (e.g., the provider is not in pain, the provider has more power). They also argue that dehumanization can be functional at times. For example, providers must learn to look at the body mechanically in order to be able to diagnose and treat the presenting issue and must reduce their empathy in order to provide care that may be painful or invasive. Trainings can focus on ways to promote the humanization of patients (e.g., eliciting background information, in addition to the presenting problem) and give providers skills to discern when empathy is useful (e.g, in routine outpatient visits) and when it should be dampened.[17]

In terms of format, training on dehumanization could consist of multiple sessions over time, incorporating instructor coaching and standardized patient interactions with feedback.[28] In addition to intensive training, patient satisfaction surveys can be a useful teaching tool, both for individual providers and for healthcare systems.[29] Our findings suggest it is important for satisfaction surveys to include measures of patient-centered care.[29] Surveys should also include questions about dehumanization, for example, the experience of being seen only as a set of symptoms, or experiences with prejudice based on race or socio-economic status.

### Limitation and future directions

One possible study limitation was selection bias. In order to participate in the interview, patients had to be literate, conversant in English, and willing to openly discuss their experiences in the healthcare system. Therefore, the results reported may underestimate the level of dehumanization experienced in the healthcare system. It is equally likely that volunteer bias may have influenced our results. Participants with highly negative experiences in healthcare may have been the most likely to volunteer for our interview. Participants who did not feel dehumanized may not have been as motivated to participate in our interview. Also, because our participants were recruited in one academic medical center, they are not representative of all populations of underserved patients, for example, those in rural settings. Thus, future samples should work to incorporate heterogeneous experiences, particularly focused on the topic of dehumanization in healthcare. Our sample was also 90% female. Perhaps women were more comfortable with the one on one, in-depth interview format. Although the themes did not differ between men and women, it is very important for future research to focus on men’s experiences in the healthcare system. However, our study is highly consistent with the literature emphasizing the importance of holistic, patient-centered care. In the future, is important for large-scale studies to examine if training providers in these areas helps to increase patient satisfaction and engage underserved patients in preventive care. Although the term patient-centered care is widely used, there is also a need to fully understand this term, which goes beyond a pleasant environment and an easy rapport described by some of our participants. Some theorists have argued that patient-centered care also includes motivating patients toward a healthier lifestyle and greater participation in their decision-making.[30] Overall, a continued focus on understanding patients’ firsthand experiences is essential to achieve the nationally recognized triple aim of improving patient experiences of care, improving population health, and reducing costs.[31]
